# The Palliative Care Service Enhancement program: specialised palliative care services for people with behaviours and psychological symptoms of dementia in Australia

**DOI:** 10.1111/psyg.13132

**Published:** 2024-05-12

**Authors:** Mustafa Atee, Thomas Morris, Daniel Whiting, Marie Alford

**Affiliations:** ^1^ The Dementia Centre HammondCare Osborne Park Western Australia Australia; ^2^ Curtin Medical School, Faculty of Health Sciences Curtin University Bentley Western Australia Australia; ^3^ Centre for Research in Aged Care, School of Nursing and Midwifery Edith Cowan University Joondalup Western Australia Australia; ^4^ Sydney Pharmacy School, Faculty of Medicine and Health The University of Sydney Sydney New South Wales Australia; ^5^ The Dementia Centre HammondCare St Leonards New South Wales Australia; ^6^ School of Public Health, Faculty of Medicine and Health The University of Sydney Sydney New South Wales Australia; ^7^ Faculty of Health University of Canberra Bruce Australian Capital Territory Australia

**Keywords:** behaviours and psychological symptoms of dementia, BPSD, dementia, Dementia Support Australia, palliative care, terminal dementia

Dementia is estimated to affect 8.4% of people aged 65 years and above in Australia.^1^ Dementia is a major and terminal neurocognitive disorder with complex care needs that requires palliative care services towards the end of life. However, palliative care models and services are lacking and only offered for 2.4% of people with dementia, compared to 75.4% for those with cancer.^2^, ^3^ Further, behaviours and psychological symptoms of dementia (BPSD) occurring at the end of life, such as terminal agitation, add considerable challenges to this population; hence additional support to individuals and caregivers is needed during this critical period to ensure comfortable death.

In Australia, Dementia Support Australia (DSA) has recognised the need for specialised palliative advice and support for people living with terminal BPSD. DSA is the national provider of BPSD support in Australia, where psychogeriatric support services are delivered free of charge to people with dementia exhibiting BPSD which impacts their care and well‐being and that of their caregivers.^4^ Funded by the Australian Government, DSA operates a number of BPSD support programs, such as the Dementia Behaviour Management Advisory Service (DBMAS) for mild to moderate BPSD and the Severe Behaviour Response Teams (SBRT) for moderate to severe BPSD.^4^ Here, we report a study that describes a DSA‐embedded pilot service that focused on optimising palliative care for people with terminal BPSD.

The Palliative Care Service Enhancement (PCSE) program was piloted by DSA during May 2021–May 2022. DSA referrals were eligible for the program if they were assessed as having terminal BPSD (e.g., agitation) as identified by a DSA medical specialist (e.g., psychogeriatrician) or through a formal assessment and confirmed by a medical practitioner (e.g., general practitioner). Eligible referrals were supported by specialist palliative and dementia‐focused consultants (comprising nursing/allied health professionals e.g., physiotherapists) who provided tailored support, advice, brokerage (resources or services, including devices, therapy or support staff, that aim to improve quality of care and deliver meaningful engagement for individuals) and liaison with external specialists. Two instruments, the Symptom Assessment Scale (SAS)^5^ and the Neuropsychiatric Inventory (NPI)^6^ were administered for palliation‐eligible DSA referrals for at least two occasions during the program. The SAS is a valid, feasible and reliable measure of distress associated with seven of the most common palliative symptoms (difficulty sleeping, appetite problems, nausea, bowel problems, breathing problems, fatigue, pain and other).^5^ Each symptom is rated on a 0–10 scale, with a score of zero categorised as distress‐free and 10 as the most severe distress score.^5^ The NPI is a validated tool for assessing the frequency and severity of BPSD and associated caregiver distress.^6^ Data were reported as counts, percents and mean (SD). This study received ethics approvals from the Human Research Committees of the University of New South Wales (HC190049), University of Sydney (2023/138), Edith Cowan University (2022–03715) and Curtin University (HRE2023‐0069).

A total of 186 DSA‐supported individuals were sub‐referred to the PCSE program. Of those, 41.9% (*n* = 78, *M*_*age*_ 84.7 (9.0) years, 49.3% female) were found eligible for palliative support. The most common dementia diagnoses for those referrals were Alzheimer's disease (*n* = 30, 39%), vascular dementia (*n* = 10, 13%), mixed dementia (*n* = 6, 7.8%) and Lewy body dementia (*n* = 3, 3.9%). The vast majority (*n* = 72, 92.3%) of PCSE‐eligible referrals were living in residential aged care homes. On average, palliative referrals were supported for 72.2 days. Following detailed onsite assessment by DSA consultants, primary causes of BPSD identified were pain (93.2%), terminal illness (56.2%), delirium (28.8%), caregiver approach (21.9%) and mood disorders (16.4%). Overall, there was a reduction of 10.4 points in the total SAS scores, 91.8% in NPI‐Caregiver distress, 84.6% in NPI‐Domains and 89.2% in NPI‐Severity scores at case closure (Fig. 1).

**Figure 1:**
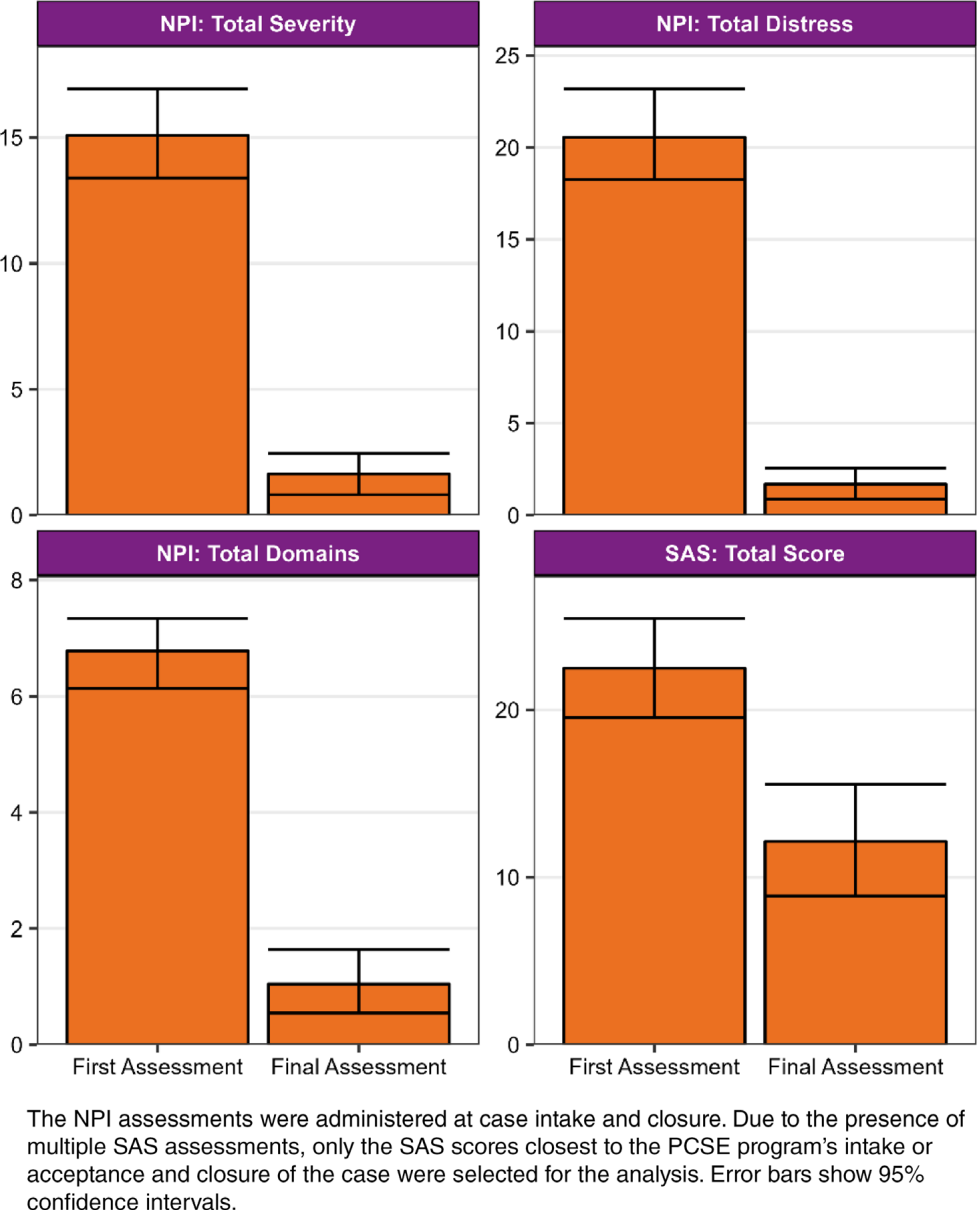
Total mean assessment scores for the Neuropsychiatric Inventory (NPI) and the Symptom Assessment Scale (SAS) during the Palliative Care Service Enhancement (PCSE) program.

There are some limitations to this study. While 41.9% of referrals were found to be eligible for palliative support, some referrals may still have been excluded. This is possibly due to the screening process used during the pilot program. Symptom and/or distress resolution or decline may occur over time without any support or intervention. For example, both appetite and fatigue are generally reduced toward the terminal phase as evident in their respective low SAS scores. This occurs as the individual becomes less interested or able to mobilise or tolerate food in the terminal phase. Further, improvement in BPSD severity in our study could be vulnerable to the time‐dependent effect previously noted by Nagata *et al*.^7^ Our study design was also limited by the lack of comparison with a control group or the standard care. Data were largely drawn from referrals from residential aged care homes, and hence the applicability of the data may not extend beyond this setting. Future research should carry out larger studies on people with BPSD from various care settings.

In conclusion, this study demonstrates the high prevalence of people with dementia with palliation needs who were seeking external BPSD support and highlights the benefits of using palliative care services for this cohort within national dementia behaviour support programs. The findings flag the urgent need for such services within the context of dementia.

## AUTHOR CONTRIBUTIONS

M.A. and T.M. conceptualised, designed and drafted the manuscript. Data analysis was completed by D.W. All authors reviewed the manuscript, provided feedback and approved the final draft for submission.

## DISCLOSURE

The authors declare they have no conflicts of interest in the research.

## Data Availability

The data that support the findings of this study will remain confidential to comply with the conditions of service provision of Dementia Support Australia (DSA).
